# Identifying acute exacerbations of chronic obstructive pulmonary disease using patient-reported symptoms and cough feature analysis

**DOI:** 10.1038/s41746-021-00472-x

**Published:** 2021-07-02

**Authors:** Scott Claxton, Paul Porter, Joanna Brisbane, Natasha Bear, Javan Wood, Vesa Peltonen, Phillip Della, Claire Smith, Udantha Abeyratne

**Affiliations:** 1 Joondalup Health Campus, Joondalup, WA Australia; 2 Genesis Care Sleep and Respiratory, Perth, WA Australia; 3 School of Nursing, Midwifery and Paramedicine, Curtin University, Bentley, WA Australia; 4 PHI Research Group, Joondalup Health Campus, Joondalup, WA Australia; 5 Institute of Health Research, University of Notre Dame, Notre Dame, WA Australia; 6 ResApp Health, Brisbane, QLD Australia; 7 School of Information Technology and Electrical Engineering, University of Queensland, Brisbane, QLD Australia

**Keywords:** Diagnostic markers, Respiratory tract diseases

## Abstract

Acute exacerbations of chronic obstructive pulmonary disease (AECOPD) are commonly encountered in the primary care setting, though the accurate and timely diagnosis is problematic. Using technology like that employed in speech recognition technology, we developed a smartphone-based algorithm for rapid and accurate diagnosis of AECOPD. The algorithm incorporates patient-reported features (age, fever, and new cough), audio data from five coughs and can be deployed by novice users. We compared the accuracy of the algorithm to expert clinical assessment. In patients with known COPD, the algorithm correctly identified the presence of AECOPD in 82.6% (95% CI: 72.9–89.9%) of subjects (*n* = 86). The absence of AECOPD was correctly identified in 91.0% (95% CI: 82.4–96.3%) of individuals (*n* = 78). The diagnostic agreement was maintained in milder cases of AECOPD (PPA: 79.2%, 95% CI: 68.0–87.8%), who typically comprise the cohort presenting to primary care. The algorithm may aid early identification of AECOPD and be incorporated in patient self-management plans.

## Introduction

Chronic obstructive pulmonary disease (COPD) is a common respiratory condition worldwide and is increasing in prevalence^1^. It is characterized by persistent respiratory symptoms due to airflow and/or alveolar abnormalities usually caused by significant exposure to noxious particles or gases^2^. Patients with COPD are susceptible to acute worsening of their symptoms with additional therapy requirements—an episode known as an acute exacerbation of COPD (AECOPD)^2^.

COPD represents a major cause of health care utilization and expense, and healthcare costs rise with each instance of AECOPD a patient experiences. Within the primary care setting in the UK, the average total annual per-patient cost of COPD management, excluding medications, was £3396 for patients experiencing two or more moderate/severe exacerbations annually, the majority of this cost being attributable to the cost of primary care consultations^3^. Similarly, a large study in the US, demonstrated a significant increase in all costs for patients with two or more exacerbations compared with the overall population of COPD patients, predominantly due to an increase in hospitalization^4^. The early identification and prevention of AECOPD such that patients no longer require hospitalization represent a critical juncture in developing a cost-effective disease management strategy.

Rapid identification of AECOPD is imperative to ensure the timely initiation of appropriate and suitable treatment^5^. It has been shown that early initiation of therapy for AECOPD reduces both exacerbation duration and the likelihood of hospitalization with an event. Delays in identifying AECOPD and thus delayed presentation to a hospital (≥24 h after symptom onset) result in a more than twofold increase in the odds of hospital admission^6^. An incorrect diagnosis can also result in inappropriate treatment with a deterioration of symptoms before the alternative diagnosis is confirmed.

Current primary-care COPD action plans allow patients to self-manage and initiate therapy for exacerbations without initial medical input^7^. This strategy depends on the patient being able to identify worsening symptoms correctly and for the symptoms to not be attributed to any co-morbidities such as asthma. A formal diagnosis of AECOPD typically requires radiology and may also require lung function tests and clinical assessment, although there are concomitant issues including inequity of access and cost. Alongside the momentum for patient-led care, is the increasing impetus toward incorporating remote-treatment technologies into primary care. This has been spurred on mainly by the reluctance of vulnerable patient populations, including those with pre-existing COPD, to present to healthcare facilities during the COVID-19 pandemic.

A reliable point of care test that can be used to rapidly and accurately diagnose COPD and exacerbations is necessary to allow early identification of an exacerbation and to allow appropriate therapy to be delivered promptly and in a manner preferred by patients.

We have previously demonstrated high diagnostic agreement of an automated algorithm in common respiratory conditions in children and the diagnosis of COPD and community-acquired pneumonia in adults^8–10^. The algorithm incorporates analysis of audio data produced during cough events. Multiple studies and a systematic review have found that computerized cough recognition technology could overcome the current limitations in the respiratory diagnostic process^11–20^. Although automated cough sound recognition technology is still relatively novel, the literature supports its efficacy and benefits, especially compared to other respiratory diagnostic methods. Traditional auscultation evaluates lower airway sounds; however, sound clarity is impeded by transmission through the chest wall. Our technology is similar to that incorporated into speech recognition technology. It evaluates a higher bandwidth of upper- and lower-airway sounds expelled via the open glottis during a coughing event. Cough events are recorded by a standard smartphone and combined with simple patient-reported clinical signs by the in-built diagnostic algorithm to provide a rapid diagnostic result without contact with participants. The addition of simple patient-reported symptoms has been found to improve the accuracy of cough analysis algorithms. Ideally, the selected clinical features are simple patient-reported symptoms that are minimally subjective and require no medical knowledge or training to identify.

In the present study, we evaluated the software algorithm’s diagnostic agreement with a comprehensive clinical diagnosis for diagnosing AECOPD in patients with known COPD.

## Results

### Demographics

**Fig. 1:**
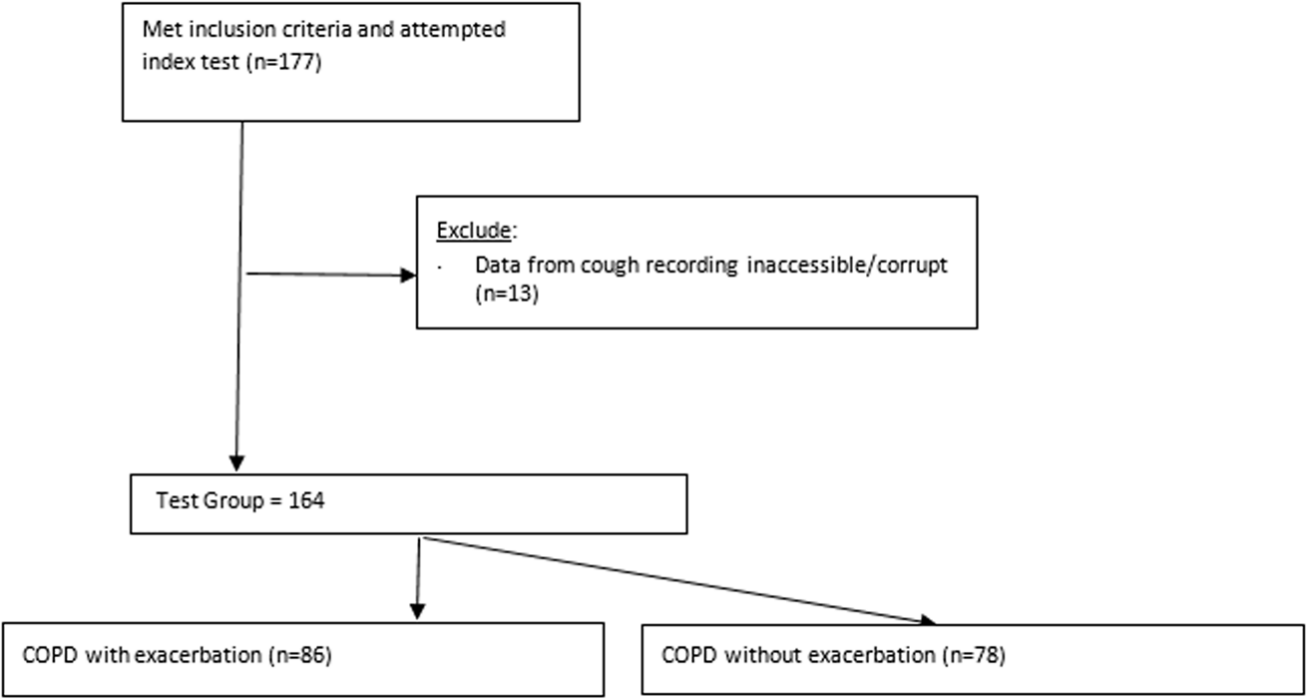
The flow of participants through the study. Depicts the recruitment and retention flow of participants in this study, leading to the final test cohort, including number with and without AECOPD.

Between December 2017 and March 2019, we enrolled 177 subjects in this prospective diagnostic accuracy study for COPD versus AECOPD study (Fig. 1). Data from cough recordings were inaccessible or corrupt for 13 subjects, leaving 164 for analysis, 78 with COPD, and 86 with AECOPD. Recruitment occurred in the emergency department, low-acuity ambulatory care, and in-patient wards of a large metropolitan hospital and the private consulting rooms of a sleep and respiratory physician in Western Australia according to defined inclusion/exclusion criteria. Diagnoses of COPD or AECOPD were per standardized clinical definitions (refer to “Methods” section).

Summary demographics are presented in Table 1. There were no differences in age (*p* = 0.744) or smoking (*p* = 0.999) between those with and without a clinical diagnosis of AECOPD. There were more females than males with AECOPD (62.8% vs. 37.2%, *p* = 0.041). A significantly higher number of participants in the AECOPD group had comorbid chronic heart failure (31.4% vs. 14.1%) (*p* = 0.009). The algorithm uses two clinical inputs, patient-reported fever and the presence of acute cough. In the AECOPD positive group (*n* = 86), 32 subjects reported the presence of fever (37%) and 60 (70%) reported acute cough.

**Table 1:** Summary demographics.

	All completed subjects (*n* = 164)	Subjects with AECOPD (*n* = 86)	Subjects without AECOPD (*n* = 78)	*p* value
Age (years)				
Mean ± SD	71.8 ± 10.2	71.6 ± 11.1	72.1 ± 9.1	*p* = 0.744
Range (min to max)	38.0–94.0	38.0–94.0	46.0–93.0	
Median (Q1, Q3)	72.0 (65.5, 79.0)	72.5 (64.0, 79.0)	72.0 (66.0, 79.0)	
Sex *n* (%)				
Male	74 (45.1%)	32 (37.2%)	42 (53.9%)	*p* = 0.041
Female	90 (54.9%)	54 (62.8%)	36 (46.2%)	
Past medical history *n* (%)				
Heart failure	38 (23.2%)	27 (31.4%)	11 (14.1%)	*p* = 0.009

*AECOPD* acute exacerbation of chronic obstructive pulmonary disease.

All subjects have underlying chronic obstructive pulmonary disease.

### Diagnostic agreement—clinical diagnosis of COPD or AECOPD (non-standard reference test) vs. algorithm (index test)

In the absence of a gold-standard test for the diagnosis of AECOPD, a clinical diagnosis was provided by a specialist respiratory physician using all available investigations and results in the medical record including the treating team’s discharge diagnosis. COPD was confirmed by spirometry for all subjects with COPD. Details of how the index test (software algorithm) was performed are provided in the “Methods” section.

The diagnostic agreement was calculated as either positive percent agreement (PPA)—the number of subjects with a positive index test result for the diagnosis of AECOPD who also have a positive clinical diagnosis (non-standard reference standard) for the same condition. Negative percent agreement (NPA) is subjects who were negative for both tests.

**Fig. 2:**
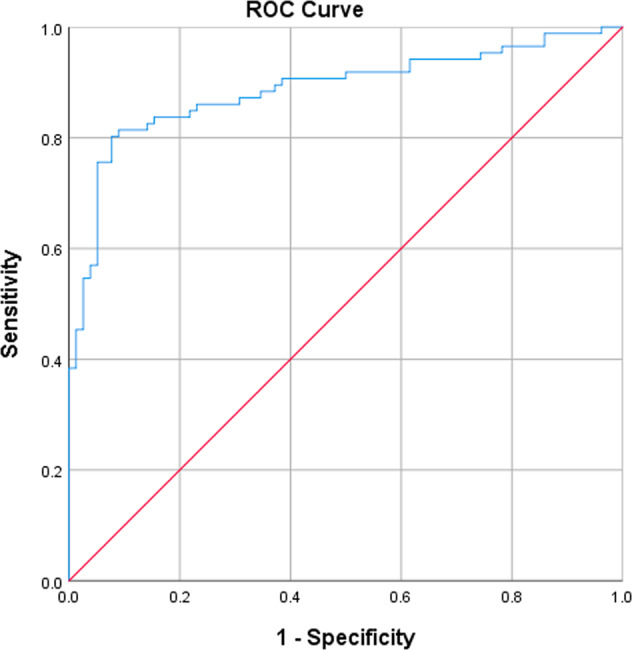
Receiver operator curve. Acute exacerbation of COPD (All ages): AUC = 0.89 (95% CI: 0.84–0.94).

**Fig. 3:**
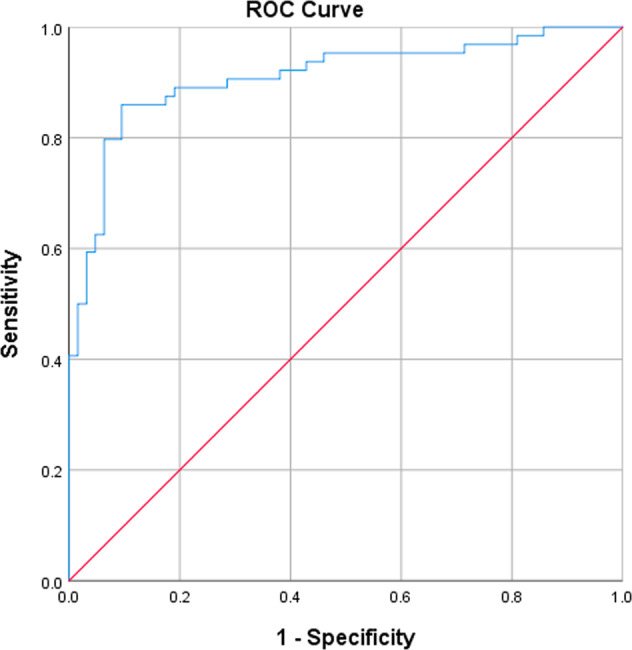
Receiver operator curve. Acute exacerbation of COPD (≥65 years): AUC = 0.91 (95% CI: 0.85–0.96).

The software algorithm demonstrated high diagnostic agreement with the clinical diagnosis (Table 2): PPA was 82.6% (95% CI: 72.9–89.9%) and NPA was 91.0% (95% CI: 82.4–96.3%). A high diagnostic agreement level was maintained in those over 65 years: PPA was 85.9% (95% CI: 75.0–93.4%) and NPA was 88.9% (95% CI: 78.4–95.4%). Plotting the receiver operator curves (ROC) curves (Figs. 2 and 3) demonstrated AUC values of 0.89 (95% CI: 0.84–0.94) and 0.91 (95% CI: 0.86–0.96) for all ages and for subjects over 65 years, respectively.

**Table 2:** Diagnostic agreement for detection of acute exacerbation of chronic obstructive pulmonary disease.

Endpoint	PPA (%) [95% CI] *n* = AECOPD positive	NPA (%) [95% CI] *n* = AECOPD negative
AECOPD, all subjects (*n* = 164)	82.6% [72.9–89.9%] *n* = 86	91.0% [82.4–96.3%] *n* = 78
AECOPD, AGED ≥ 65 YEARS (*n* = 127)	85.9% [75.0–93.4%] *n* = 64	88.9% [78.4–95.4%] *n* = 63

*PPA* positive percent agreement, *NPA* negative percent agreement, *AECOPD* acute exacerbation of chronic obstructive pulmonary disease.

We then evaluated the performance of the algorithm by the AECOPD severity category assigned using the CRB-65 criteria. CRB-65 assigns a grade between 0 and 4 with a score of 0–1 predicting a low risk of 30-day mortality (suitable for community management), a score of 2 predicting a moderate risk of 30-day mortality (standard hospital admission), and a score of 3–4 predicting a high risk of 30-day mortality (requiring urgent hospital treatment)^21^. Of the 86 subjects with AECOPD, 20 had a score of 0, 52 had a score of 1, and 14 had a score of 2. There were no subjects with scores of 3 or 4. Subjects with scores of 0 or 1 (*n* = 72), were correctly identified as having AECOPD in 79.20% (95% CI: 68.0–87.8%) of cases. All subjects with scores of 2 (*n* = 14) were correctly identified; however, the small number of subjects in this group precluded formal accuracy reporting.

## Discussion

Our study has shown that a smartphone-based algorithm was accurate in identifying patients with known COPD experiencing an exacerbation (AECOPD) with a PPA of 82.6% (95% CI: 72.9–89.9%) and NPA of 91.0% (95% CI: 82.4–96.3%). Accuracy was maintained in subjects aged greater than 65, in those with comorbid heart failure and AECOPD-positive subjects with milder exacerbations. The area under the ROC curves were 0.89 (95% CI: 0.84–0.94) for all ages and 0.91 (95% CI: 0.86–0.96) for subjects over 65 years, respectively.

Current international diagnostic criteria for AECOPD are based upon clinical judgment and are reliant upon ready access to supporting investigations and clinician experience. Diagnostic discrimination is particularly problematic when the AECOPD phenotype is mild, as is frequently encountered in the primary-care setting. At present, patient self-management of COPD is encouraged by the use of written action plans and is effective at reducing respiratory-related hospitalization^22^. Written action plans frequently guide the patient to initiate therapy with oral steroids and antibiotics without direct clinician involvement^7,22^. The use of oral steroids is common in respiratory disease, particularly for patients with severe disease or AECOPD. However, steroid use is associated with significant adverse effects, including type-2 diabetes, obesity, osteoporosis, all of which contribute to the morbidity of COPD and should be used with caution.

A key component in the deployment of self-management plans is the requirement of the patient to self-recognize their AECOPD, a task which many patients may be unable to perform. Around two-thirds of patients cannot recognize that the worsening of at least one key symptom (dyspnea, sputum amount, and color) represents an exacerbation of their COPD, are confused over the use of the term exacerbation, and misinterpret the presence and severity of their AECOPD^23–25^. To minimize this difficulty, we only included three simple, easily understood patient-reported clinical features in our algorithm (age, fever, and presence of a new cough).

The AECOPD algorithm sits within a suite of other related algorithms that diagnose other potential respiratory diseases simultaneously. The three clinical features we use are not additive to those required for the other algorithms giving a total of only six features combined.

Various diagnostic scoring techniques have been proposed to assist in identifying AECOPD in patients with known COPD. However, their validity depends upon an accurate description and capture of patient symptoms. For example, the Clinical COPD Questionnaire, when employed weekly to discriminate AECOPD from stable COPD, demonstrated a sensitivity of 62.5% and specificity of 82.0%, (AUC = 0.75). However, the questionnaire was reliant on patient reporting of symptoms such as sputum volume and sputum color and reported poor compliance with the requested weekly monitoring^26^. The use of similar questionnaires, such as the COPD assessment questionnaire—a tool designed initially for COPD detection which determines AECOPD likelihood by evaluating the change in impairment score from week to week—is limited by its ability to capture changes experienced over the preceding week rather than acutely presenting symptoms^27^. A similar study reported high sensitivity and specificity (96%/98%) but required daily reporting of symptoms by patients as indicated by at least two consecutive days of suggestive symptoms with follow-up of suspected AECOPD by a pulmonologist^28^.

Another approach is to incorporate remote spirometry alongside patient-reported symptoms into the diagnosis of AECOPD^29^. Though requiring a large number of daily symptom-based questions, this process allowed for early detection of AECOPD in the majority (73%) of cases and reduced the hospitalization rates. The approach suffers from the same limitations: patients are required to identify and interpret symptoms by themselves and provide data over days to identify respiratory symptoms trends. The reliance of self-management plans for COPD on subjective inputs from patients limits their utility unless more objective diagnostic tools can be incorporated into them. There is potential for the algorithm we have developed to be incorporated into a self-management plan for AECOPD as it provides a rapid, on-the-spot result, without requiring a prolonged, retrospective comparison of symptoms to baseline. In addition, the algorithm requires simple patient-reported symptoms (age and presence of fever or cough during this illness) plus five recorded cough sounds and does not require clinical expertise to interpret the inputted signs (presence of acute cough/fever and age).

Our algorithm’s accuracy was maintained in the older age group, where the frequency of comorbidities is likely to be greater. Heart failure is a common co-morbidity with COPD and causes similar symptoms, including exertional breathlessness and nocturnal cough/dyspnea. Diagnosis of COPD and AECOPD in this group is complicated by the ventilatory defects exhibited by patients with heart failure, which obscure the diagnostic airflow limitation characteristic of COPD. In some cases, patients with heart failure can recognize the symptoms of their AECOPD but may avoid or delay therapy because of the risk of side effects^24^. As would be expected, there was an increased prevalence of chronic heart failure in patients with clinically diagnosed AECOPD in our study. Despite this, our algorithm demonstrated high diagnostic accuracy in this group.

In remote or Telehealth assessments, clinical assessment and auscultation are nearly impossible, and obtaining vital signs may require an assistant at the remote location. Additionally, many patients with COPD are frail and have low mobility with difficulty attending facilities, particularly if their exacerbations are frequent. The possibility of remote monitoring is attractive to patients as it reduces the risk of nosocomial infection with more severe and potentially antibiotic-resistant infections. Limitations in previous studies evaluating COPD diagnosis via a telehealth interface have identified a high attrition rate due to technical issues/lacking necessary equipment – problems which may be pertinent in an older population as usually afflicted by COPD. In contrast, the algorithm we have developed requires only the use of a standard smartphone and a phone connection to convey the diagnostic result to a clinician, allowing the potential for its deployment as a component of a Telehealth platform or as a standalone device. The maintenance of high diagnostic accuracy in milder cases of AECOPD lends further support to our algorithm’s potential use in community management scenarios.

The subjects included in our study were predominantly Caucasian with smoking-related COPD and thus may not be generalizable to instances where the underlying COPD has a different etiology. Additionally, although the algorithm interface is simple to use, in this study all inputs to the smartphone were made by experienced operators who also assisted the patient in the recording of the coughs. Usability and safety studies of the algorithm delivered via a smartphone have been performed by ResApp Health (Australia) and reported for EU and TGA regulatory submissions. These studies included identifying the key hazard-related use scenarios; ergonomic analysis; heuristic analysis; handedness testing; aberrant behavior testing; and usability. The application was found to be easily used by patients without safety concerns.

The study was conducted at a single site in a clinical environment and the majority (84%) of subjects who presented with known AECOPD were categorized as mild. This reflects a situation where the technology could be deployed however as the app is operator- and site-independent the potential use scenarios are broader and allows for patients to use the tool at home.

In conclusion, we have developed a smartphone-based algorithm using simple patient-reported characteristics and audio analysis of cough events that demonstrates high diagnostic agreement for the diagnosis of acute exacerbations of COPD in patients with known COPD. Diagnostic accuracy was maintained across AECOPD severity levels and in older patients. In comparison to other AECOPD diagnostic tools, the diagnostic result is virtually instantaneous and is not reliant upon monitoring symptom decline over several days nor upon subjective interpretation of patient symptoms. The algorithm has the potential to improve the diagnosis of AECOPD in patients presenting to health care facilities, in remote and resource-limited situations, and in circumstances where presentation to healthcare facilities is not possible.

## Methods

### Ethical approval

Informed written consent was obtained from all participants, and the Ramsay Healthcare Human Research and Ethics Committee, Western and South Australia, approved the study (REF: 1501).

### Development of the algorithm (Index Test)

Subjects were recruited for this study as part of a more extensive program developing diagnostic algorithms for pediatric and adult respiratory conditions (BreatheEasy: ACTRN12618001521213). There were two discrete, collected cohorts recruited to develop each algorithm: a training set and a testing set. The program’s goal was to create a set of algorithms to be run simultaneously that would sit on a standard smart device to diagnose and differentiate the common respiratory diseases seen in adults. Targeted disease groups included isolated upper respiratory disease, lower respiratory disease (any cause), COPD, COPD exacerbations, acute asthma, pneumonia, and subjects with no respiratory disease.

Each algorithm consists of a combination of sound analysis and simple, patient-derived clinical symptoms and characteristics. The aim is to use the smallest number of clinical features that would encompass all of the targeted conditions while still ensuring they could be easily identified and reported by non-clinicians (patients or non-expert health providers). Our Pneumonia algorithm includes fever, acute cough, productive cough, and age; our acute asthma algorithm has age, presence of acute or productive cough, fever, and wheeze; and our COPD detection algorithm has age; smoking pack-years, acute cough, and fever. These three conditions use a total of only six, patient-derived inputs^9,10^.

From January 2016 to November 2017 we recruited 1228 subjects >12 years into an independent training cohort, consisting of patients presenting to our study locations with any of the following features: rhinorrhoea, sore throat, sneezing (during this illness), cough (acute, chronic or productive), wheeze, fever, shortness of breath or new-onset hoarse voice (during this illness). Subjects without respiratory disease were also recruited. Specialist clinicians confirmed the subject’s clinical diagnosis by reviewing all medical notes, treatments, investigations (including spirometry and radiology where available), and clinical course. A final diagnosis was only applied after the subject had completed all medical care to ensure an accurate diagnosis. From this cohort, algorithms were developed for the selected targeted conditions.

A proprietary software application was developed for this work. Recorded audio data were analyzed offline on Apple Macintosh computers using proprietary C++ software developed via a machine learning approach. The entire process was automated.

We developed an automatic cough detector that identifies cough sounds using Time Delay Neural Network, identifying Mel Frequency Cepstral Coefficients (MFCC) from the continuous audio stream^13,30^ The detector calculates features forming a feature vector which is used to classify audio segments as either cough or non-cough by a machine-learning classifier. The classifier was trained on a dataset of manually selected cough and non-cough events. The audio segments are combined to form completed cough events.

The diagnostic algorithm was developed using the extraction of mathematical features from cough samples, with selected features used to build a classifier model^11,31,32^.

To refine the algorithms, we used the initial 1228 cough sound datasets and the corresponding clinical diagnosis. The analysis consisted of picking all cough events from each audio recording, calculating MFCC from the cough audio, and feeding them, along with selected clinical features, into a logistic regression model to identify the targeted disease. An optimal model was designed using a combination of feature selection and cross-fold validation on the training dataset. These optimal models were locked prior to use in prospective diagnostic accuracy studies. Each disease’s model was developed independently using diagnoses for that particular disease only.

The diagnostic algorithm for the detection of AECOPD was developed for a prospective validation study. We used the clinical definitions in Table 3 to define the presence of COPD and AECOPD. An extensive list of symptoms was initially analyzed for inclusion in the algorithm including dyspnoea/shortness of breath, presence of productive cough, wheeze, upper respiratory symptoms (runny nose, sneezing, and stuffiness), lethargy, nausea/vomiting, loss of appetite, voice change, new cough (<7 days), productive cough, chronic cough, fever, smoking history and age. Based on test performance in the training set, patient-reported fever or a new cough during this illness (Y/N) and patient age were selected as the input features for the final, optimized AECOPD algorithm. Selecting these features did not add to the total already used in our other algorithms. The features are not sufficient to accurately diagnose AECOPD on their own, however, were selected as they improved the performance of the algorithm while being simple, generally understood, and likely to be reliably reported by patients. Optimal diagnostic performance was seen when the audio-analysis was included in addition to the patient-reported features.

**Table 3:** Study case definitions.

COPD	- Respiratory symptoms consistent with COPD and history of smoking (>10 pack-years)/environmental exposure AND: ○ If spirometry performed, then FEV1/FVC2 < 0.7 on the best test (after bronchodilator) OR - If spirometry not performed, then a previous physician-diagnosis of COPD.
AECOPD	- ALL OF: ○ Met COPD case definition (as above), ○ Worsening symptoms of shortness of breath (SOB), cough; - Signs and symptoms of acute respiratory tract infection - Treating team diagnosis of AECOPD confirmed by specialist review.

*COPD* chronic obstructive pulmonary disease, *FEV1/FVC* forced expiratory volume in 1 s/forced vital capacity, *AECOPD* acute exacerbation of chronic obstructive pulmonary disease.

Once the optimal model was developed, the algorithm was locked. An independent testing set was prospectively recruited, from the same locations and with the same inclusion criteria as the development cohort.

### Prospective diagnostic agreement study

Subjects were approached if they presented to the participating site with signs or symptoms of respiratory disease or presented to specialist rooms for a lung function test. The present analysis set only included patients from the BreatheEasy prospective validation set (*n* = 850) with diagnosed COPD or AECOPD as per the definition in Table 3 (*n* = 229, 147 exacerbated, 82 not exacerbated). Subjects were excluded if they were on ventilatory support, had a terminal disease, were medically unstable, had a medical contraindication to providing a voluntary cough (e.g., severe respiratory distress, eye; chest or abdominal surgery within 3 months; a history of pneumothorax or had structural airway disease. All participants provided written informed consent.

AECOPD severity was scored using the CRB-65—a score of between 0 and 4, which assigns 1 point for each of confusion, increased respiratory rate (≥ 30/min), decreased blood pressure (SBP < 90 mmHg or DBP ≤ 60 mmHg), and age ≥ 65 years. CRB-65 is a clinical prediction tool used to grade AECOPD severity as indicated by 30-day mortality^21^.

A Clinical Diagnosis of COPD or AECOPD (non-standard reference test) was reached as follows. A research nurse performed a clinical assessment (including auscultation and other respiratory symptoms) of the subject and took a medical history, including current medications. Subjects were asked to complete a spirometry test according to standard methodology^33^. A specialist clinician reviewed the medical file for each subject, including the results of any radiology/laboratory tests performed, and assigned a clinical diagnosis based on the definitions listed in Table 3.

Where the audio data was not available, subjects were excluded from further analysis. When a clinical diagnosis had been assigned to all subjects, the database was locked, and a separate operator ran the software to ensure blinding was maintained.

Subjects were asked to provide five coughs. Cough sounds were recorded using a smartphone (iPhone6), held by a research nurse, approximately 50 cm away from the subject at an angle of 45-degree angle to the direction of the airflow from the subjects’ mouth. The recording was undertaken in a clinical setting but was performed in a manner to ensure minimal external noise was recorded. If the subject could not provide five coughs as determined by the cough sound recording software, the subject was excluded from further analysis.

Cough recordings and clinical examination were performed at the same time.

### Statistical analysis

Power calculations were derived as follows. Based on expected positive and NPA greater than 85% from the training program, to obtain a superiority endpoint of 75% (lower bound 95% CI of maximum width ±0.10) a minimum of 48 cases were required for each disease.

The primary study endpoint was defined as a PPA and NPA of the index test with the non-standard reference standard, with 95% confidence intervals calculated using the method of Clopper–Pearson. The probability of a positive clinical diagnosis was calculated for each subject by the final classifier model and used as the decision thresholds in the derived ROC. The analysis was performed for the total cohort and for subjects over 65 years. Demographic details are presented as means, medians, and quartiles with standard deviations and compared using paired *t* tests. All data were analyzed using Stata 14.1 (StataCorp, College Station, TX).

### Reporting summary

Further information on research design is available in the Nature Research Reporting Summary linked to this article.

## Supplementary information

Reporting Summary

## Data Availability

The datasets supporting the conclusion of this article are available at reasonable request from P.P. The cough recordings are not available but will be uploaded as an educational tool at the conclusion of the Breathe Easy development program in 2022.

## Code availability

The underlying codes are the property of ResApp Health and are not available.
