# Supplementary material for: Identifying acute exacerbations of chronic obstructive pulmonary disease using patient-reported symptoms and cough feature analysis
Source: NPJ Digit Med. 2021 Jul 2;4:107. doi: 10.1038/s41746-021-00472-x (PMC8253790; doi:10.1038/s41746-021-00472-x)
Supplement: Supplementary file 1 — Reporting Summary [file 41746_2021_472_MOESM1_ESM.pdf]

nature research

Corresponding author(s): Paul Porter

Last updated by author(s): 28 May 2021

Reporting Summary

Nature Research wishes to improve the reproducibility of the work that we publish. This form provides structure for consistency and transparency in reporting. For further information on Nature Research policies, see our Editorial Policies and the Editorial Policy Checklist.

Statistics

For all statistical analyses, confirm that the following items are present in the figure legend, table legend, main text, or Methods section.

n/a	Confirmed	
☐	☒	The exact sample size (*n*) for each experimental group/condition, given as a discrete number and unit of measurement
☐	☒	A statement on whether measurements were taken from distinct samples or whether the same sample was measured repeatedly
☐	☒	The statistical test(s) used AND whether they are one- or two-sided<br>*Only common tests should be described solely by name; describe more complex techniques in the Methods section.*
☒	☐	A description of all covariates tested
☒	☐	A description of any assumptions or corrections, such as tests of normality and adjustment for multiple comparisons
☐	☒	A full description of the statistical parameters including central tendency (e.g. means) or other basic estimates (e.g. regression coefficient) AND variation (e.g. standard deviation) or associated estimates of uncertainty (e.g. confidence intervals)
☐	☒	For null hypothesis testing, the test statistic (e.g. *F*, *t*, *r*) with confidence intervals, effect sizes, degrees of freedom and *P* value noted<br>*Give P values as exact values whenever suitable.*
☒	☐	For Bayesian analysis, information on the choice of priors and Markov chain Monte Carlo settings
☐	☒	For hierarchical and complex designs, identification of the appropriate level for tests and full reporting of outcomes
☒	☐	Estimates of effect sizes (e.g. Cohen's *d*, Pearson's *r*), indicating how they were calculated

Our web collection on statistics for biologists contains articles on many of the points above.

Software and code

Policy information about availability of computer code

Data collection	Microsoft Excel 2016
Data analysis	Stata 14.1 (StataCorp, College Station, TX)

For manuscripts utilizing custom algorithms or software that are central to the research but not yet described in published literature, software must be made available to editors and reviewers. We strongly encourage code deposition in a community repository (e.g. GitHub). See the Nature Research guidelines for submitting code & software for further information.

Data

Policy information about availability of data

All manuscripts must include a data availability statement. This statement should provide the following information, where applicable:

- Accession codes, unique identifiers, or web links for publicly available datasets
- A list of figures that have associated raw data
- A description of any restrictions on data availability

The datasets supporting the conclusion of this article are available on reasonable request and with reciprocal ethical approval from the corresponding author. The cough recordings are not available but will be uploaded as an educational tool at the conclusion of the Breathe Easy development program in 2022.

Field-specific reporting

Please select the one below that is the best fit for your research. If you are not sure, read the appropriate sections before making your selection.

☒ Life sciences ☐ Behavioural & social sciences ☐ Ecological, evolutionary & environmental sciences

For a reference copy of the document with all sections, see nature.com/documents/nr-reporting-summary-flat.pdf

Life sciences study design

All studies must disclose on these points even when the disclosure is negative.

Sample size	Power calculations were derived as follows. Based on expected positive and negative percent agreement greater than 85% from the training program, to obtain a superiority endpoint of 75% (lower bound 95% CI of maximum width ±0.10) a minimum of 48 cases were required for each disease.
Data exclusions	Data from cough recordings were inaccessible or corrupt for 13 subjects and excluded
Replication	This is a diagnostic accuracy study. We report the initial development of the algorithm with this DA being the "replication study"
Randomization	Convenience sample. All subjects were administered both tests.
Blinding	The diagnosing team and algorithm testing were independent from each other as was the statistician who performed the analysis. When a clinical diagnosis had been assigned to all subjects, the database was locked, and a separate operator ran the software to ensure blinding was maintained.

Reporting for specific materials, systems and methods

We require information from authors about some types of materials, experimental systems and methods used in many studies. Here, indicate whether each material, system or method listed is relevant to your study. If you are not sure if a list item applies to your research, read the appropriate section before selecting a response.

Materials & experimental systems

n/a	Involved in the study	
☒	☐	Antibodies
☒	☐	Eukaryotic cell lines
☒	☐	Palaeontology and archaeology
☒	☐	Animals and other organisms
☐	☒	Human research participants
☐	☒	Clinical data
☒	☐	Dual use research of concern

Methods

n/a	Involved in the study	
☒	☐	ChIP-seq
☒	☐	Flow cytometry
☒	☐	MRI-based neuroimaging

Human research participants

Policy information about studies involving human research participants

Population characteristics	164 subject, mean age 71.8 years, 45% male, 23.2% preexisting heart failure. All with diagnosed COPD.
Recruitment	A dedicated research nurse approached potential participants. Written informed consent was obtained prior to the study commencement.
Ethics oversight	Ramsay Healthcare Human Research and Ethics Committee, Western and South Australia, approved the study (REF: 1501)

Note that full information on the approval of the study protocol must also be provided in the manuscript.

Clinical data

Policy information about clinical studies

All manuscripts should comply with the ICMJE guidelines for publication of clinical research and a completed CONSORT checklist must be included with all submissions.

Clinical trial registration	ACTRN12618001521213
Study protocol	Protocol is available on https://anzctr.org.au/Trial/Registration/TrialReview.aspx?id=375939&isClinicalTrial=False

Data collection	Recruitment occurred in the emergency department, low-acuity ambulatory care and in-patient wards of a large metropolitan hospital and the private consulting rooms of a sleep and respiratory physician in Western Australia
Outcomes	The primary study endpoint was defined as a PPA and NPA of the index test (algorithm) with the non-standard reference standard (clinically diagnosed AECOPD), with 95% confidence intervals calculated using the method of Clopper-Pearson. The probability of a positive clinical diagnosis was calculated for each subject by the final classifier model and used as the decision thresholds in the derived Receiver Operator Curve. The analysis was performed for the total cohort and for subjects over 65 years. Demographic details are presented as means, medians, and quartiles with standard deviations and compared using paired t-tests.
